# Prevalence of anatomical variations at the suboccipital (V3) segment of the vertebral artery: a systematic review

**DOI:** 10.1007/s00234-023-03223-9

**Published:** 2023-10-25

**Authors:** Bukola R. Omotoso, Rohen Harrichandparsad, Lelika Lazarus

**Affiliations:** 1 Discipline of Clinical Anatomy, School of Laboratory Medicine and Medical Sciences, College of Health Sciences, University of KwaZulu-Natal, Westville Campus, Private Bag X54001, Durban, 4000 South Africa; 2 Department of Neurosurgery, School of Clinical Medicine, Nelson R Mandela School of Medicine, College of Health Sciences, University of KwaZulu-Natal, Durban, South Africa

**Keywords:** Suboccipital vertebral artery, Persistent first intersegmental artery, Fenestration, Posterior inferior cerebellar artery, Computed tomography angiography

## Abstract

**Background and objective:**

A recent meta-analysis on the incidence of iatrogenic injury to the VA has revealed that patients with variant anatomy are more prone to iatrogenic injury. Therefore, this review is designed to investigate the incidence of variations in the suboccipital component of the vertebral artery in different population groups according to the available literature.

**Methods:**

This systematic review was conducted according to PRISMA guidelines (Preferred Reporting Items for Systematic Reviews and Meta-Analyses). The review is based on a comprehensive and extensive search of PubMed, Google Scholar, and ResearchGate. The following search terms were used: “vertebral artery” AND “suboccipital segment” AND “anomalies/anatomical variations of the V3 segment.” Reference lists of all extracted articles were also extensively searched for references to any further relevant publications.

**Results:**

A total of 17 papers met the inclusion criteria. The 17 studies corresponded to a total of 10,820 patients. A persistent first intersegmental artery was registered in 1.8% (197 out of 10,820) of the patients. Extradural PICA origin was observed in 1.6% (175 out of 10,820) of the patients. Fenestration was detected in 0.7% (72 out of 10,820) of the patients.

**Conclusion:**

The authors summarize the incidence of vascular variation at the suboccipital segment of the VA in different population groups across the Asian, European, American, and African continents. Awareness of the extent of possible anatomical variation will help interpret radiographs, which will enhance the identification of vascular pathologies and reduce the risk of iatrogenic injury.

## Introduction

Vertebral artery (VA) injuries during surgical interventions around the atlantoaxial region constitute a potentially catastrophic complication that may result in permanent neurological deficit or even death [1, 2]. The rates of injury range from 1.7 to 9.0% [2–4]. The segment of the VA located in the atlantoaxial region is the suboccipital (V3) segment. The V3 segment is the most anatomically complicated as the artery undergoes a series of bends to form proximal and distal loops while passing through the transverse foramen of the axis and atlas vertebrae. Although mild tortuosity is a variation in the course of the VA usually reported at the V1 and V2 segments. However, loop formation distinguishes the V3 segment from other segments of the artery. This feature of the V3 segment predisposes it to iatrogenic injury when performing skull base surgical procedures and instrumentation at the atlantoaxial region [5]. The V3 segment is subdivided into three portions for description: the vertical portion ascends through the transverse foramen of C2 and C1; the horizontal portion extends from the transverse foramen of C1 and courses in the VA groove on the upper surface of the posterior arch of the atlas; and an oblique portion extends from the groove to the point of penetration of the posterior atlanto-occipital membrane [6, 7]. The VA typically gives off muscular branches (also known as the suboccipital artery of Salmon) in the suboccipital region [8]. These small branches are not always present and are rarely seen on angiograms.

Apart from the aforementioned standard anatomical description, reports of variant vascular anatomy such as fenestration (FEN), persistent first intersegmental artery (FIA), hypoplasia, and incidence of the posterior inferior cerebellar artery (PICA) arising from the V3 segment also contribute to the complexity of this segment [9, 10]. A recent meta-analysis on the incidence of iatrogenic injury to the VA has revealed that patients with variant anatomy are more prone to iatrogenic injury [11]. This is because variant arteries are often situated in an unanticipated position. Therefore, the present study has been designed to review the available literature on the anatomic variations peculiar to this segment in different population groups and their incidences.

## Materials and methods

### Search strategy

**Fig. 1:**
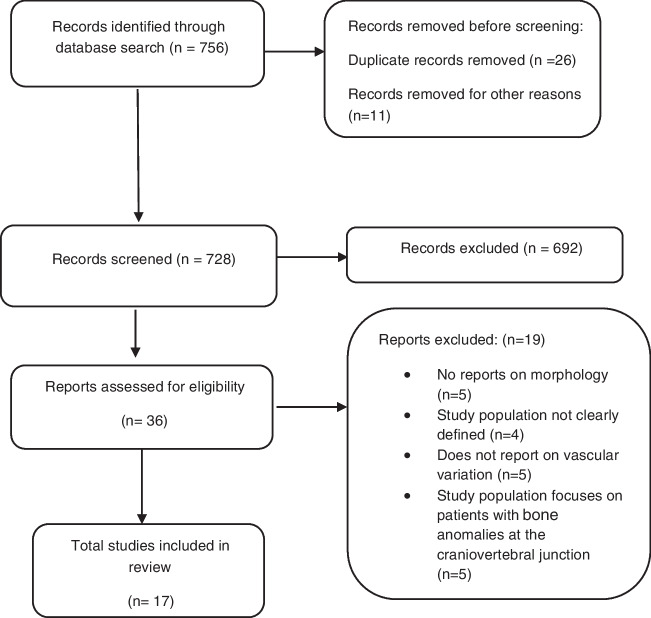
PRISMA flowchart showing the identification and evaluation of studies included in the systematic review

This systematic review was conducted according to PRISMA guidelines (Preferred Reporting Items for Systematic Reviews and Meta-Analyses) [12] (Fig. 1). This review is based on a comprehensive and extensive search of PubMed, Google Scholar, and ResearchGate. The following search terms were used: “vertebral artery” AND “suboccipital segment” AND “anomalies/anatomical variations of the V3 segment.” Reference lists of all extracted articles were also extensively searched for references to any further relevant publications. Primary studies that addressed the review question were included. Case studies, reviews, letters to the editors, conference abstracts, or studies containing incomplete or irrelevant data were excluded. The protocol for this review was registered in PROSPERO (Registration ID CRD42020173699).

### Criteria for study selection

This review includes English language angiographic studies (including digital subtraction angiography, magnetic resonance angiography, and computed tomography angiography, large series studies excluding case reports and reviews). In every single article, the reported variation in morphology at the suboccipital segment of the VA was recorded in the study population without bone anomalies at the craniovertebral junction (CVJ).

### Inclusion criteria

The following are the inclusion criteria: all research articles on anatomical variations, morphology, and morphometry of the suboccipital segment of the VA and studies in English language, angiographic studies, or studies combining cadaveric with angiographic.

### Exclusion criteria

The following are the exclusion criteria: research articles with no evidence of knowledge of anatomical variations and morphology of the suboccipital segment of the VA.

### Eligibility assessment

The initial title screening was done by the principal investigator. Included studies at the title screening stage were exported to a library on EndNote reference manager (EndNote X9) for abstract and full article screening, both of which were guided by the eligibility criteria. The EndNote library was shared with a second screener (LL) for abstract and full article screening. Any discrepancies in the screeners’ results of abstract screening were resolved through discussion until an agreement was reached. Discrepancies in full article screening results were resolved by a third screener (RH). Records that were duplicated and did not meet the eligibility criteria were excluded.

### Data extraction

Details on characteristics of the included studies (names of the first author and year of publication, study sample, modality of the study, sample size, the incidence of FIA, PICA, and FEN) were extracted (Table 1).

**Table 1:** Studies reporting on the prevalence of anatomical variation at the suboccipital (V3) segment of the vertebral artery (VA)

					Anatomical variations (number of patients)			Total percentage of variation	Conclusion (recommendation of routine preoperative angiography)
Author (year)	Sample size	Sex M/F	Study population	Type of study	FIA	PICA	FEN		
Tokuda et al. 1985 [13]	300	–	Japanese	DSA	2	2	3	2.3%	Not recommended
Hong et al. 2008 [14]	1013	446/567	South Korean	CTA	48	2	6	5.5%	Recommended
Yamaguchi et al. 2008 [15]	140	69/71	Japanese	CTA	4	23	1	20%	Recommended
Duan et al. 2010 [16]	98	66/32	Chinese	CTA	3	0	3	6%	Recommended
Uchino et al. 2012 [9]	2739	1615/1124	Japanese	MRA	87	30	25	5%	Recommended
O'Donnell et al. 2014 [17]	975	591/384	American	CTA	1	4	1	0.6%	Not recommended
Wakao et al. 2014 [18]	387	269/118	Japanese	CTA	7	5	5	4.4%	Recommended
Fortuniak et al. 2016 [10]	1800	968/832	Polish	CTA	0	11	3	0.8%	Not recommended
Hong et al. 2016 [19]	123	–	South Korean	CTA	3	1	0	3.6%	Recommended
Kim 2016 [20]	546 314	–	South Korean	CTA	7 8	11 9	2 2	3.7% 6%	Recommended
Vaněk et al. 2017 [21]	511	328/183	Czech	DSA	2	21	1	4.7%	Recommended
Zhu et al. 2018 [22]	678	-	Japanese	DSA	6	10	12	4%	Recommended
Isaji et al. 2018 [23]	142	76/66	Japanese	CTA	4	27	1	22.5%	Recommended
Zhang et al. 2018 [24]	200	105/95	Chinese	CTA	7	0	0	3.5%	Recommended
Xu et al. 2018 [25]	100	61/39	Chinese	CTA	2	1	5	7%	Not recommended
Arslan et al. 2019 [26]	200	112/88	Turkish	CTA	1	2	0	1.5%	Recommended
Omotoso et al., 2021	554	307/247	South African	MDCTA	5	16	2	4.2%	Not Recommended
Total	10,820				197	175	72		
Average of variants					11.6	10.3	4.2		

According to the article by Kim [20], two different sample sizes (546, 314) from CT angiography and cervical spinal CT angiography were used, so we separated the two sample sizes.

*Keys: M* male, *F* female, *FIA* persistent first intersegmental artery, *PICA* posterior inferior cerebellar artery, *FEN* fenestration.

### Bias assessment

The AQUA (Anatomical Quality Assurance) Tool was used to appraise the methodological quality (risk of bias) of the studies [27]. AQUA is a tool used to examine the quality of the included studies by assessing five different domains: study objectives and subject characteristics, study design, methodology characterization, descriptive anatomy, and reporting results. These five domains are all assessed in terms of risk of bias. Each category was assessed as having low risk, high risk, or unclear. Overall, studies were evaluated as either having a low risk or being at high risk of bias [27].

## Results

### Description of included studies and patient’s characteristics

Most of the included studies are from the Asian continent (12/17) (Table 1). Regarding the sample size, a large proportion of the study population were male (for the studies that reported sex differences) (Table 1). The majority of studies used CTA as the imaging modality of choice, and most studies recommend routine preoperative angiography to prevent iatrogenic injury to the VA (Table 1). A total of 17 papers met the inclusion criteria. The vascular variation reported includes FIA, extradural PICA origin, and FEN, with each paper reporting at least two or more of the identified anatomical variants. The articles ranged in date from 1985 to 2021 and included angiographic studies from different populations. The 17 studies corresponded to a total of 10,820 angiograms. FIA was registered in 1.8% (197/10,820) of the patients. Extradural PICA origin was observed in 1.6% (175/10,820) of the patients. Fenestration was detected in 0.7% (72/10,820) of the patients. Results are presented in Table 1.

### The methodological quality of the included studies

All included studies (17) were of good quality as the risk of bias was judged “low” following assessment with AQUA Tool. In the case where any of the signaling questions on the AQUA Tool answered “NO,” indicating potential for bias, all authors reached a consensus regarding the eligibility of the study.

## Discussion

Adequate information about anatomical variation can influence the choice of procedure for the treatment of deformities and injuries at the CVJ. The V3 segment is susceptible to injury at the point of transition from V2 to V3, particularly within the C-2 groove when inserting a C1-2 transarticular screw or when placing C-2 pars screws [28, 29]. Authors have also identified potential sites of injury at different portions of the V3 segment in cadaveric samples [7]. The advent of computed tomography angiography (CTA), a unique non-invasive imaging modality, has improved the diagnosis and detection of vascular variation at the V3 segment. Its uniqueness lies in its ability to show details of vascular anatomy and its relationship with the atlas and the axis vertebrae. Recent advances in CTA have demonstrated that vascular structures can easily be depicted, which has caused some level of reduction in the use of invasive examinations such as digital subtraction angiography (DSA) [30]. According to some institutions, DSA remains the gold standard for assessing the vascular system. However, it is an invasive technique with potential hitches such as permanent neurological complications as a direct consequence of the procedure [30, 31]. In a recent meta-analysis comparing CTA with DSA, the latter technique was traditionally regarded as the gold standard for evaluating and treating vascular pathology. More recently, CTA has been viewed as a technique of choice. However, the most appropriate choice between these imaging techniques remains debatable [31]. For instance, the American Heart and Stroke Association lists DSA as a class I recommendation in their guidelines for managing aneurysmal subarachnoid hemorrhage. However, the American College of Radiology’s Appropriateness Criteria list CTA ahead of DSA but gives equal scores for both [32]. CTA is also occasionally used as a complementary imaging technique to conventional angiography for follow-up [33]. In addition, studies have shown that CTA allows reliable evaluation of intracranial arterial pathology, including aneurysms, stenosis, and occlusion [34]. Post-processed images in the 3D workstation of CTA and the raw images provide better details and opportunities for a detailed description of the presence of anatomical variation [18]. This is also confirmed in our review, as most of the studies evaluating anatomical variations at the suboccipital segment of the VA used CTA as the imaging modality (Table 1). However, CTA is not without its shortcomings. In addition to the high-cost implication, the use of ionizing radiation and iodinated contrast media has been associated with adverse events such as contrast allergy, nephrotoxic effects, neurological injury, and stroke, especially in patients with chronic kidney disease [17, 30, 35].

Embryologically, the VA develops from the fusion of six primitive cervical intersegmental arteries (CIAs). The seventh CIA persists to form the subclavian artery and the point of origin of the VA, while the first CIA is also referred to as the proatlantal artery [36]. As reported by Luh and co-authors, the horizontal portion of the V3 segment develops specifically from the anastomosis between the proatlantal artery and CIAs [37]. This theory suggests that the vertical portion of the V3 segment may have developed together with the V1 and V2 segments from the primitive CIAs. This is possible due to the dynamic and unique embryogenesis of the VA compared with any other arterial vessel [38].

**Fig. 2:**
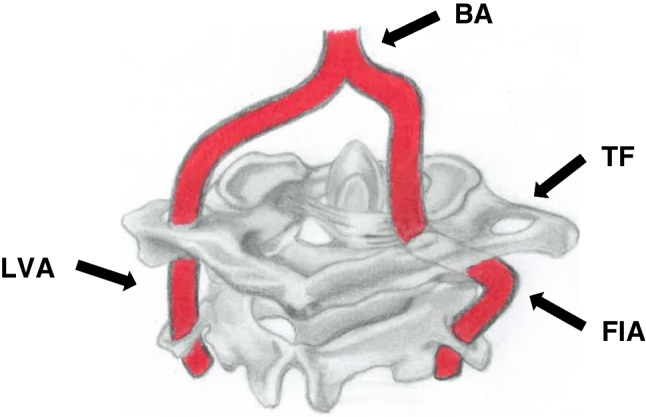
A schematic diagram of the suboccipital segment (posterior view) of the bilateral vertebral artery shows the persistent first intersegmental artery (FIA) of the right vertebral artery. The left vertebral artery (LVA) had a typical course through the transverse foramen of the atlas and axis vertebrae. TF, transverse foramen of C1 (atlas); BA, basilar artery

Anatomical variations have been identified as a clinically significant risk factor for VA injury, especially during the posterior approach to the V3 segment [11, 39, 40]. In a recent multi-center study on the epidemiology of iatrogenic VA injury, the overall incidence of injury was reported as 0.08%, with posterior fixation surgery around C1-2 having the highest risk of injury (1.35%) [41]. Other previous studies have also reported that the incidence of iatrogenic injury may be as high as up to 8% in posterior fixation surgery [42], higher than 0.5% registered during anterior cervical spine surgery [11]. Iatrogenic injury during cervical spine surgery can cause catastrophic bleeding, permanent neurological deficits such as stroke, and even death [11, 41]. The most reported vascular variation at the suboccipital segment of the VA is FIA (Table 1, Fig. 2). An abnormal intradural course of the V3 segment of the VA through the spinal canal between the vertebral body of C2 and C1 has been previously defined with different terminologies such as; an “aberrant VA coursing intradurally at the C2 level” [20] and “FIA” [9, 10]. The latter is the most frequently used. The prevalence of this variation reported by individual authors ranges between 0.01 and 3.2% [9, 17, 26]. Similarly, the overall incidence was 1.8% in this review (197/10820) (Table 1).

**Fig. 3:**
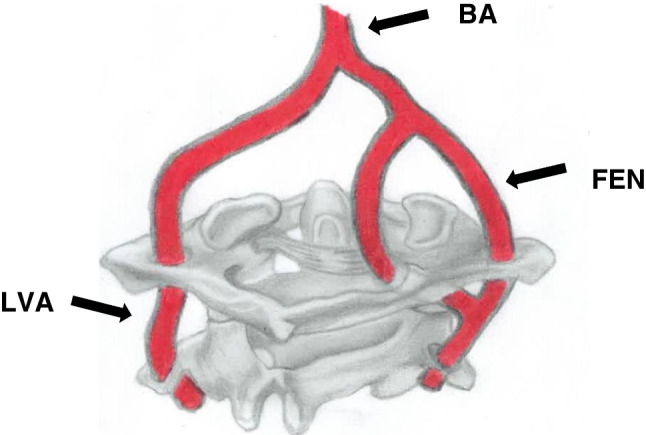
A schematic diagram of the suboccipital segment (posterior view) of the bilateral vertebral artery shows fenestration of the right vertebral artery. The left vertebral artery (LVA) had a typical course. BA, basilar artery

**Fig. 4:**
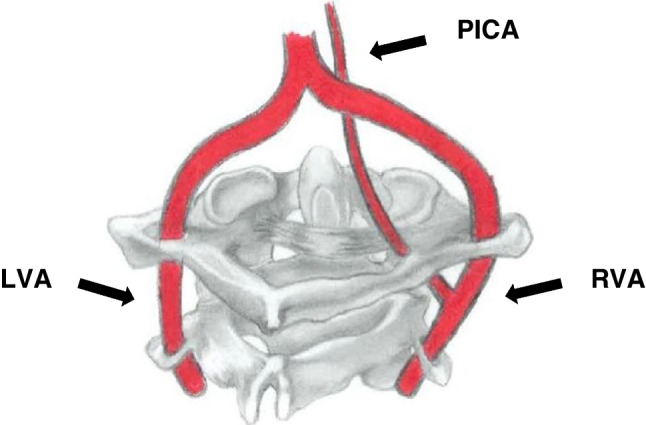
A schematic diagram of the suboccipital segment (posterior view) of the bilateral vertebral artery shows the posterior inferior cerebellar artery (PICA) arising from the suboccipital segment of the right vertebral artery (RVA). The left vertebral artery (LVA) had a typical course. BA, basilar artery

Fenestration is the least reported variation in the V3 segment, 0.7% (72/10820) (Table 1, Fig. 3). FEN can be described as a luminal division of an artery with a common origin into two separate and parallel channels anywhere along its course, which rejoin distally [43]. A recent study proposed two terminologies for FEN at the suboccipital segment of the VA: a C2 segmental type of the VA associated with a normal VA or an aberrant VA with an intradural course at the C2 level associated with a normal VA [20]. However, the term “fenestration” was not acknowledged in the suggested names. In our own opinion, although fenestration sometimes coexists with FIA, it should be described separately for lucidity. The prevalence of FEN ranges from 0.01 to 1.3% [17, 18]. The incidence observed in the present review is within the range reported by previous studies. PICA is the principal branch of the VA, and it typically originates from the intracranial part of the vertebral artery (4^th^ segment). However, due to numerous embryonic vessels involved in forming the VA and its branches, PICA sometimes emerges from the V3 part (Fig. 4). We observed an incidence rate of 1.6% (175/10820) in the present review. An abnormal course of the VA or its PICA branch below the C1 arch may predispose the arteries to iatrogenic injuries during drilling, tapping, and insertion of lateral mass screws [26].

In this review, the highest incidence of variation was registered in the Asian population at 5.5% (374/6780) (Table 2). This is followed by a recent report from South Africa representing the African population [4.2% (23/554)] [44]. The incidences reported in the European [1.6% (41/2511)] and the American population [0.6% (6/975)] were similar, although slightly higher in the European population (Table 2). It should be noted that the reported incidence from this review is skewed since most available studies in the literature are from the Asian population, followed by the European population (Table 1). We found one study each representing the American and African populations [17, 44]. Although the theory is not well understood, authors have hypothesized genetic and environmental factors as the likely cause of differences in the incidence of VA variations [17, 21, 45]. A recent longitudinal study of the vertebrobasilar system focusing on Caucasian twins has suggested that the morphology of the vertebrobasilar arterial system is a function of genetic factors in combination with some environmental effects [46]. This important factor may have contributed to the regional differences, as reported in this review. However, the disparity in the incidence may also be due to underreporting from other population groups apart from the Asian population. It is important to note that the prevalence of variation in the included study is for the normal population without CVJ anomalies. The incidence of variation is usually higher in patients with congenital bone anomalies at the CVJ, such as atlantoaxial dislocation [25, 47].

**Table 2:** Studies reporting on variation at the suboccipital (V3) segment of the vertebral artery (VA) summarized according to different population groups

Population	Total sample size	Total reported variation	Percentage
Asian	6780	374	5.5%
European	2511	41	1.6%
American	975	6	0.6%
African	554	23	4.2%

In some instances, the morphology of the variant VA and osseous relationship at the CVJ is incompatible with screw placement. In the presence of FIA, insertion of a C1-2 transarticular screw may not be appropriate; C1 superior lateral mass may be the best alternative [48]. On the other hand, in the presence of FEN and PICA, a transarticular screw may be the best option, as placing a C-2 pars screw should be avoided [48]. Since most of this variation occurs unilaterally, preoperative imaging will be useful to determine the anomalous side. It has been suggested that it is preferable to commence dissection from the normal side during surgical intervention [5]. Vascular variation at the V3 segment can be present without any clinical symptoms and is sometimes symptomatic when it coexists with a disease condition. For instance, FIA has been associated with cervical cord myelopathy and other clinical presentations such as cervical pain, occipital neuralgia, and accessory nerve palsy [49]. Also, FEN has been associated with an aneurysm due to irregularities in the vascular walls of the fenestrated segment [50]. In addition to anatomical studies from different population groups, studies highlighting the incidence of iatrogenic injury from clinical series may give a better idea of the necessity of preoperative CTA in different population groups.

## Study limitation

Because only a few of the included manuscripts presented data on morphometry, there was no sufficient data to perform a meta-analysis of pooled data. Regarding morphology, most of the studies were conducted in Asia. This may skew the result, and it may not be easy to generalize results beyond Asia. Hence, we grouped the included studies into Asian, European, American, and African and commented on the differences.

## Conclusion

The authors summarize the incidence of vascular variation at the suboccipital segment of the VA in different population groups across the Asian, European, American, and African continents (Table 2). Although most available data are from the Asian population, other regions are also represented. This review will contribute to the knowledge of evidence-based anatomy. Regarding the low prevalence of variation at the V3 segment in most of the population groups except Asia as mapped out in this review, we do not recommend routine preoperative CTA before the surgical intervention or endovascular treatment of vascular pathologies. Alternatively, a non-contrast CT scan may be considered. While such scans do not demonstrate detailed vascular anatomy, they may suggest the typical and variable course of the VA. When an anatomical variation is suspected in the course of the VA, CTA may be required for clarification. However, when the prevalence is high in a population group such as the Asian, routine preoperative CTA should be considered to prevent iatrogenic injury. Awareness of the extent of possible anatomical variation will help interpret radiographs, which will enhance the identification of vascular pathologies and reduce the risk of iatrogenic injury.

## Data Availability

All data generated or analysed during this study are included in this article without restriction.

## References

[1] Akinduro OO, Baum GR, Howard BM (2016). Neurological outcomes following iatrogenic vascular injury during posterior atlanto-axial instrumentation. Clin Neurol Neurosurg.

[2] Vergara P, Bal JS, Hickman Casey AT (2012). C1–C2 posterior fixation: are 4 screws better than 2?. Oper Neurosurg.

[3] Elliott RE, Tanweer O, Boah A (2014). Comparison of screw malposition and vertebral artery injury of C2 pedicle and transarticular screws: meta-analysis and review of the literature. Clin Spine Surg.

[4] Liang M-L, Huang M-C, Cheng H (2004). Posterior transarticular screw fixation for chronic atlanto-axial instability. J Clin Neurosci.

[5] Menon RG, Prasad GL (2015). Decoding the V3 segment of the vertebral artery. J Neurology India.

[6] George B, Cornelius J (2001). Vertebral artery: surgical anatomy. Oper Tech Neurosurg.

[7] Ulm AJ, Quiroga M, Russo A (2010). Normal anatomical variations of the V3 segment of the vertebral artery: surgical implications. J Neurosurg Spine.

[8] Tubbs RS, Shah NA, Sullivan BP (2009). Surgical anatomy and quantitation of the branches of the V2 and V3 segments of the vertebral artery. J Neurosurg Spine.

[9] Uchino A, Saito N, Watadani T (2012). Vertebral artery variations at the C1–2 level diagnosed by magnetic resonance angiography. Neuroradiology.

[10] Fortuniak J, Bobeff E, Polguj M (2016). Anatomical anomalies of the V3 segment of the vertebral artery in the Polish population. Eur Spine J.

[11] Guan Q, Chen L, Long Y (2017). Iatrogenic vertebral artery injury during anterior cervical spine surgery: a systematic review. J World neurosurgery.

[12] Page MJ, Moher D, Bossuyt PM (2021). PRISMA 2020 explanation and elaboration: updated guidance and exemplars for reporting systematic reviews. BMJ.

[13] Tokuda K, Miyasaka K, Abe H (1985). Anomalous atlantoaxial portions of vertebral and posterior inferior cerebellar arteries. Neuroradiology.

[14] Hong JT, Lee SW, Son, BC et al (2008) Analysis of anatomical variations of bone and vascular structures around the posterior atlantal arch using three-dimensional computed tomography angiography. J Neurosurg Spine 230–236. 10.3171/SPI/2008/8/3/230 10.3171/SPI/2008/8/3/230 18312074

[15] Yamaguchi S, Eguchi K, Kiura Y (2008). Posterolateral protrusion of the vertebral artery over the posterior arch of the atlas: quantitative anatomical study using three-dimensional computed tomography angiography. J Neurosurg Spine.

[16] Duan S, He H, Lv S, Chen L (2010). Three-dimensional CT study on the anatomy of vertebral artery at atlantoaxial and intracranial segment. Surg Radiol Anat.

[17] O'Donnell CM, Child ZA, Nguyen Q (2014). Vertebral artery anomalies at the craniovertebral junction in the US population. Spine.

[18] Wakao N, Takeuchi M, Nishimura M (2014). Vertebral artery variations and osseous anomaly at the C1–2 level diagnosed by 3D CT angiography in normal subjects. Neuroradiology.

[19] Hong JT, Kim IS, Kim JY (2016). Risk factor analysis and decision-making of surgical strategy for V3 segment anomaly: significance of preoperative CT angiography for posterior C1 instrumentation. Spine J.

[20] Kim MS (2016). Developmental anomalies of the distal vertebral artery and posterior inferior cerebellar artery: diagnosis by CT angiography and literature review. Surg Radiol Anat.

[21] Vaněk P, Bradáč O, De Lacy P (2017). Vertebral artery and osseous anomalies characteristic at the craniocervical junction diagnosed by CT and 3D CT angiography in normal Czech population: analysis of 511 consecutive patients. Neurosurg Rev.

[22] Zhu SW, Yang Y, Liu YG (2018). Anatomical features and clinical significance of radiculomuscular artery variants involving the suboccipital segment of vertebral artery. Clin Neuroradiol.

[23] Isaji T, Yasuda M, Kawaguchi R (2018). Posterior inferior cerebellar artery with an extradural origin from the V3 segment: higher incidence on the nondominant vertebral artery. J Neurosurg Spine.

[24] Zhang H, Chai W, Wang S (2018). Persistent first intersegmental artery (PFIA) visualized by three-dimensional computed tomography angiography in Chinese population. Int J Surg.

[25] Xu S, Ruan S, Song X (2018). Evaluation of vertebral artery anomaly in basilar invagination and prevention of vascular injury during surgical intervention: CTA features and analysis. Eur Spine J.

[26] Arslan D, Ozer MA, Govsa F (2019). Surgicoanatomical aspect in vascular variations of the V3 segment of vertebral artery as a risk factor for C1 instrumentation. J Clin Neurosci.

[27] Tomaszewski KA, Henry BM, Kumar Ramakrishnan P (2017). Development of the Anatomical Quality Assurance (AQUA) checklist: guidelines for reporting original anatomical studies. Clin Anat.

[28] Neo M, Sakamoto T, Fujibayashi S (2005). The clinical risk of vertebral artery injury from cervical pedicle screws inserted in degenerative vertebrae. Spine.

[29] Gluf WM, Schmidt MH, Apfelbaum RI (2005). Atlantoaxial transarticular screw fixation: a review of surgical indications, fusion rate, complications, and lessons learned in 191 adult patients. J Neurosurg Spine.

[30] Akgun V, Battal B, Bozkurt Y et al (2013) Normal anatomical features and variations of the vertebrobasilar circulation and its branches: an analysis with 64-detector row CT and 3T MR angiographies. The scientific world journal. 2013. 10.1155/2013/620162 10.1155/2013/620162 PMC3759058 24023533

[31] Uricchio M, Gupta S, Jakowenko N (2019). Computed tomography angiography versus digital subtraction angiography for postclipping aneurysm obliteration detection: a meta-analysis. Stroke.

[32] Salih M, Moore JM, Ogilvy CS (2021). Computed tomography angiography versus digital subtraction angiography as a primary diagnostic tool in nontraumatic subarachnoid hemorrhage: cost-effectiveness analysis study. World Neurosurg.

[33] Chen CJ, Tseng YC, Lee TH (2004). Multisection CT angiography compared with catheter angiography in diagnosing vertebral artery dissection. AJNR Am J Neuroradiol.

[34] Kovač JD, Stanković A, Stanković D (2014). Intracranial arterial variations: a comprehensive evaluation using CT angiography. Med Sci Monit: Int Med J Exp Clin Res.

[35] Solomon RJ, Natarajan MK, Doucet S (2007). Cardiac Angiography in Renally Impaired Patients (CARE) study: a randomized double-blind trial of contrast-induced nephropathy in patients with chronic kidney disease. Circulation.

[36] Padget DH (1948). The development of the cranial arteries in the human embryo. J Contrib Embryol.

[37] Luh G, Dean B, Tomsick T (1999). The persistent fetal carotid-vertebrobasilar anastomoses. AJR Am J Roentgenol.

[38] Giuffre R, Sherkat S (1999). The vertebral artery: developmental pathology. J Neurosurg Sci.

[39] DeCarvalho SA, Abd-El-Barr MM, Groff MW (2019) Vascular complications in cervical spine surgery (anterior and posterior approach), in Complications in neurosurgery Elsevier 314–319.

[40] Hsu WK, Kannan A, Mai HT (2017). Epidemiology and outcomes of vertebral artery injury in 16 582 cervical spine surgery patients: an AOSpine North America Multicenter Study. Glob Spine J.

[41] Lee C-H, Hong JT, Kang DH (2019). Epidemiology of iatrogenic vertebral artery injury in cervical spine surgery: 21 multicenter studies. World Neurosurg.

[42] Nourbakhsh A, Yang J, Ziran B (2015). An unusual course of the vertebral artery posterior to the nerve root in the inter-transverse space: a cadaveric study. Patient Saf Surg.

[43] Omotoso B, Harrichandparsad R, Moodley I (2021). Fenestration of the vertebrobasilar junction detected with multidetector computed tomography angiography. Folia Morphol.

[44] Omotoso BR, Harrichandparsad R, Satyapal KS (2021). Radiological anatomy of the suboccipital segment of the vertebral artery in a Select South African population. Eur J Anat.

[45] Sikka A, Jain A (2012) Bilateral variation in the origin and course of the vertebral artery. Anat Res Int. 2012. 10.1155/2012/580765 10.1155/2012/580765 PMC3375040 22720161

[46] Szalontai L, Jokkel Z, Horvath T (2021). Are the morphological indices of the vertebrobasilar system heritable? A twin study based on 3D reconstructed models. Medicina.

[47] Yamazaki M, Okawa A, Furuya T (2012). Anomalous vertebral arteries in the extra-and intraosseous regions of the craniovertebral junction visualized by 3-dimensional computed tomographic angiography: analysis of 100 consecutive surgical cases and review of the literature. J Spine.

[48] Lin X, Zhu H-J, Xu Y (2021). Prevalence of Vertebral artery anomaly in upper cervical and its surgical implications: a systematic review. Eur Spine J.

[49] Takahashi T, Tominaga T, Hassan T (2003). Cervical cord compression with myelopathy caused by bilateral persistence of the first intersegmental arteries: case report. Neurosurgery.

[50] Kubo M, Hacein-Bey L, Varelas PN (2005). Ruptured saccular aneurysm of distal vertebral artery fenestration managed with Guglielmi detachable coils and intraventricular tissue plasminogen activator. Surg Neurol.

